# Necrotizing periodontal disease: Oral manifestation of COVID‐19

**DOI:** 10.1111/odi.13462

**Published:** 2020-06-22

**Authors:** Jay Patel, Julian Woolley

**Affiliations:** ^1^ School of Dentistry University of Leeds Leeds UK; ^2^ King's College Hospital London UK

**Keywords:** bacterial co‐infection, coronavirus disease 2019, COVID‐19, necrotizing periodontal disease

We read with interest the series of cases reported by Martín Carreras‐Presas, Amaro Sánchez, López‐Sánchez, Jané‐Salas, and Somacarrera Pérez (2020). We believe that the described oral vesiculobullous manifestations were suggestive of coronavirus disease 2019 (COVID‐19) co‐infections, which, at present, are overlooked and poorly understood (Cox, Loman, Bogaert, & O'Grady, 2020). Increased disease severity and mortality among individuals with respiratory viral infections are often attributed to subsequent bacterial co‐infections, accounting for approximately 95% of deaths during the 1918 Spanish flu pandemic (Morens, Taubenberger, & Fauci, 2008).

We predict a spontaneous rise in the prevalence of acute periodontal lesions, particularly necrotizing periodontal disease (NPD), in accordance with the increase in COVID‐19 confirmed cases. The etiology of NPD lesions may be associated with bacterial co‐infections occurring intra‐orally in COVID‐19 patients. Metagenomic analyses of those infected with severe acute respiratory syndrome coronavirus 2 (SARS‐CoV‐2) frequently detect abnormally high bacterial reads of *Prevotella intermedia* in addition to common pathogenic genera implicated in the onset and progression of oral diseases such as *Streptococci, Fusobacterium, Treponema,* and *Veillonella* (Chakraborty, 2020). *P. intermedia* is considered a major etiological bacterial species for several acute periodontal lesions, which, alongside *Fusobacterium* and *Treponema* species, constitute a large proportion of the microbiota present in NPD lesions (Herrera, Retamal‐Valdes, Alonso, & Feres, 2018). NPDs are more prevalent in patients with HIV (Herrera et al., 2008). In a mechanistically similar way, SARS‐CoV‐2 infection may predispose individuals to NPDs through bacterial co‐infection propagated by *P. intermedia* (Patel & Sampson, 2020). We present a case of a patient attending the King's College Hospital with NPD and suspected COVID‐19:

A 35‐year‐old female patient without any relevant medical history attended the acute dental emergency setting reporting fever, halitosis, intense gingival pain, and bleeding. The fever presented 3 days prior to any oral symptoms. Examination revealed that she was apyrexic. She presented bilateral submandibular lymphadenopathy, and an intra‐oral examination confirmed severe halitosis, generalized erythematous and edematous gingivae, and necrotic interdental papillae in both the maxillary and mandibular labial sextants. Bleeding was evident from the gingival sulcus without provocation, and there was no detectable attachment loss. A clinical diagnosis of necrotizing gingivitis was made. Although COVID‐19 was suspected, it was not possible to provide testing at the time. The patient was prescribed 400mg metronidazole three times daily for 5 days and 0.12% chlorhexidine mouthwash twice daily for 10 days. Following national guidance on COVID‐19, the patient was advised to return home immediately to self‐isolate for 7 days. The patient was called 5 days later and described complete resolution of her oral symptoms and fever. Notably, this patient's oral and COVID‐19 suspected symptoms had resolved following the antibiotic regimen, strengthening the role of bacterial co‐infections in COVID‐19 severity.

There is an urgent need to study co‐infections in COVID‐19 patients and encourage clinicians to diagnose these conditions early, owing to their contribution to mortality and heightened disease severity in historic pandemics of respiratory viral infections. Routine intra‐oral examinations for COVID‐19 patients should be provided across healthcare disciplines.

## CONFLICT OF INTEREST

None to declare.

## AUTHOR CONTRIBUTION

**Jay Patel:** Conceptualization; Data curation; Writing‐original draft; Writing‐review & editing. **Julian Woolley:** Writing‐original draft; Writing‐review & editing.
